# Pre-α-pro-GDNF and Pre-β-pro-GDNF Isoforms Are Neuroprotective in the 6-hydroxydopamine Rat Model of Parkinson's Disease

**DOI:** 10.3389/fneur.2018.00457

**Published:** 2018-06-20

**Authors:** Anna-Maija Penttinen, Ilmari Parkkinen, Merja H. Voutilainen, Maryna Koskela, Susanne Bäck, Anna Their, Christopher T. Richie, Andrii Domanskyi, Brandon K. Harvey, Raimo K. Tuominen, Liina Nevalaita, Mart Saarma, Mikko Airavaara

**Affiliations:** ^1^HiLIFE Unit, Institute of Biotechnology, University of Helsinki, Helsinki, Finland; ^2^Division of Pharmacology and Pharmacotherapy, Faculty of Pharmacy, University of Helsinki, Helsinki, Finland; ^3^National Institute on Drug Abuse, National Institutes of Health, Baltimore, MD, United States

**Keywords:** neurotrophic factors, neurodegeneration, GDNF, splice variant, alternative splicing, tyrosine hydroxylase, dopamine

## Abstract

Glial cell line-derived neurotrophic factor (GDNF) is one of the most studied neurotrophic factors. GDNF has two splice isoforms, full-length pre-α-pro-GDNF (α-GDNF) and pre-β-pro-GDNF (β-GDNF), which has a 26 amino acid deletion in the pro-region. Thus far, studies have focused solely on the α-GDNF isoform, and nothing is known about the *in vivo* effects of the shorter β-GDNF variant. Here we compare for the first time the effects of overexpressed α-GDNF and β-GDNF in non-lesioned rat striatum and the partial 6-hydroxydopamine lesion model of Parkinson's disease. GDNF isoforms were overexpressed with their native pre-pro-sequences in the striatum using an adeno-associated virus (AAV) vector, and the effects on motor performance and dopaminergic phenotype of the nigrostriatal pathway were assessed. In the non-lesioned striatum, both isoforms increased the density of dopamine transporter-positive fibers at 3 weeks after viral vector delivery. Although both isoforms increased the activity of the animals in cylinder assay, only α-GDNF enhanced the use of contralateral paw. Four weeks later, the striatal tyrosine hydroxylase (TH)-immunoreactivity was decreased in both α-GDNF and β-GDNF treated animals. In the neuroprotection assay, both GDNF splice isoforms increased the number of TH-immunoreactive cells in the substantia nigra but did not promote behavioral recovery based on amphetamine-induced rotation or cylinder assays. Thus, the shorter GDNF isoform, β-GDNF, and the full-length α-isoform have comparable neuroprotective efficacy on dopamine neurons of the nigrostriatal circuitry.

## Introduction

Originally purified from a rat glioma cell line, glial cell-derived neurotrophic factor (GDNF) was shown to promote differentiation and survival of rat midbrain dopamine neurons, increase outgrowth of neurites and dopamine uptake *in vitro* (1). Moreover, GDNF stimulated the formation of new axon terminals in dopamine neurons (2). These findings led to increased interest in GDNF's therapeutic potential for Parkinson's disease (PD), in which the progressive degeneration of midbrain dopamine neurons in substantia nigra pars compacta (SNpc) and their projections to striatum (caudate nucleus and putamen) is causing major motor disturbances, such as tremor and postural instability (3). Indeed, in animal models of PD, GDNF has been shown to protect the dopaminergic nigrostriatal pathway from 6-OHDA or MPTP-induced degeneration when administered as a protein or gene therapy (4–7), and to restore the dopaminergic phenotype (i.e., striatal dopaminergic markers, such as tyrosine hydroxylase (TH) and the dopamine level) of the pathway after the degeneration in rodent and non-human primate models of PD (3, 8–11).

**Figure 1:**
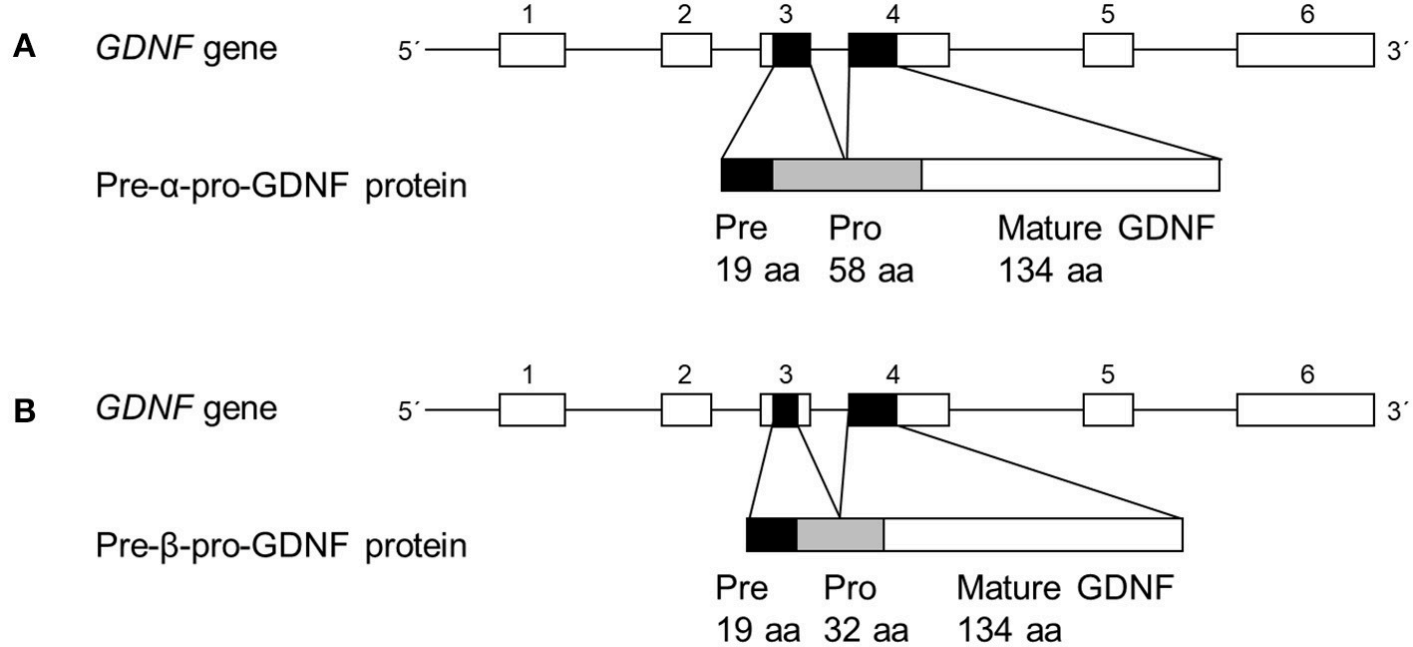
Organization of main human GDNF splice isoforms. **(A,B)** In *GDNF* gene line represents introns and boxes represent exons (not in scale). Black boxes represent protein coding areas. Pre-α-pro-GDNF isoform has a full-length 58 amino acid pro-region, whereas the pre-β-pro-GDNF has shorter 32 amino acid pro-region.

The human GDNF gene consists of six exons and the rodent GDNF gene of three exons (12–14) (Figure 1). The alternative splicing site in the third exon produces two conserved splice isoforms; full-length pre-α-pro-GDNF (α-GDNF) and the shorter pre-β-pro-GDNF (β-GDNF), which has a deletion of 26 amino acids (GKRPPEAPAEDRSLGRRRAPFALSSDS) in the pro-region (12–16) (Figure 1). The deletion does not interfere with the proteolytic cleavage site, and both isoforms are cleaved to mature GDNF. The pre-region is cleaved off in the endoplasmic reticulum and the pro-region mainly in the secretory vesicles (1, 16, 17). The pro-region has been suggested to play a role in the folding and secretion of GDNF (18). *In vitro*, both isoforms are secreted from the cells upon overexpression but in drastically different manner. α-GDNF and the corresponding mature GDNF are secreted constitutively while β-GDNF and its corresponding mature GDNF are secreted activity-dependently (17). Furthermore, the isoforms have different localization patterns inside the cells: α-GDNF is mainly localized in the Golgi complex, whereas β-GDNF is localized in secretogranin II (scgII)- and Rab3A-positive vesicles of the regulated secretory pathway (17). Despite these differences in localization and secretion, the two major splice isoforms, α-GDNF and β-GDNF, are expressed in the same tissues, but in varying proportions (14–16). Interestingly, β-GDNF mRNA expression is present at relatively high levels during brain development when neuronal contacts are formed (15).

GDNF is functional as a homodimer, stabilized by a disulfide-bond (19). It exerts its functions via binding first to a lipid raft-resident glycosylphosphoinositol-anchored GDNF receptor α (GFRα), followed by formation of a heterohexameric complex with two Ret (rearranged during transfection) receptors (20). Alternatively, the signaling is initiated by GDNF-GFRα via NCAM (21) or syndecan-3 (22). The exact pro-survival mechanism of GDNF is not known, but activation of Ret can initiate several signaling cascades, of which the mitogen activated protein (MAP) kinase and phosphoinositositide-3-kinase (PI3K) pathways have been suggested to play a role in the survival promoting actions (23).

Although GDNF is a widely studied trophic factor, and its potential as a therapeutic agent for neurodegenerative diseases is well established including human clinical trials for Parkinson's disease, there are only few studies about the biology of β-GDNF. All previous studies have focused on the effects and properties of α-GDNF, whereas the biological effects of the shorter β-isoform are still largely unknown. This is the first study to compare the effects of the two major GDNF isoforms in non-lesioned striatum as well as in the 6-hydroxydopamine (6-OHDA) rat model of PD. We report here the effects of β-GDNF to be comparable to the effects of α-GDNF on the dopaminergic phenotype of the nigrostriatal dopamine neurons. In non-lesioned striatum, both GDNF isoforms increased the density of dopamine transporter (DAT)-immunoreactive striatal fibers 3 weeks after viral vector delivery, but only α-GDNF increased the use of contralateral paw in the cylinder test at the same time point. Four weeks later, overexpression of both isoforms downregulated TH. However, the isoforms equally protected the TH-immunoreactive cell bodies in SNpc against 6-OHDA-induced degeneration.

## Materials and methods

### Generation of pscAAV-CMV-pre-α-pro-GDNF and pscAAV-CMV-pre-β-pro-GDNF constructs

To produce the self-complementary AAV (scAAV) vectors expressing human pre-α-pro-GDNF and pre-β-pro-GDNF, the cDNA fragments encoding human pre-α-pro-GDNF and pre-β-pro-GDNF were produced by PCR using pAAV-pre-α-pro-GDNF and pAAV-pre-β-pro-GDNF (17) as a template accordingly. PCR was performed with Phusion Hot-Start polymerase (ThermoFisher Scientific, Waltham, MA). PCR products were purified and digested by BamHI and NotI restriction enzymes (ThermoFisher Scientific, Waltham, MA) and ligated into a pscAAV-CMV vector using T4 DNA ligase (ThermoFisher Scientific, Waltham, MA). The plasmid pscAAV-CMV was obtained by cutting out the eGFP insert from pscAAV-CMV-eGFP using BamHI and NotI restriction sites. Both cloned constructs were verified by DNA sequencing. Primers used for cloning of pre-α-pro-GDNF and pre-β-pro-GDNF into pscAAV-CMV were forward 5′-TAGGATCCATGAAGTTATGGGATGTCGTGG-3′ containing BamHI restriction site and reverse 5′-TAGCGGCCGCTCAGATACATCCACACCTTTTA-3′ containing NotI restriction site.

The self-complementary AAV vectors, scAAV-pre-α-pro-GDNF, scAAV-pre-β-pro-GDNF and scAAV-CMV-eGFP were packaged as serotype 1 (24), then purified and titered as described previously (25). The titers for the vectors were scAAV1-CMV-eGFP 7.40 × 10^13^ vg/ml, scAAV1-CMV-pre-α-pro-GDNF 2.14 × 10^12^ vg/ml, and scAAV1-CMV-pre-β-pro-GDNF 1.73 × 10^12^ vg/ml, respectively. AAV vector work was conducted by the Optogenetics and Transgenic Technology Core, NIDA IRP, NIH, Baltimore MD, USA.

### Animals

The experiments were carried out in accordance with the 3R principles of the EU directive 2010/63/EU on the care and use of experimental animals, and local laws and regulations [Finnish Act on the Protection of Animals Used for Scientific or Educational Purposes (497/2013) and Government Decree on the Protection of Animals Used for Scientific or Educational Purposes (564/2013)]. All animal procedures were reviewed and approved by the national Animal Experiment Board of Finland (License number ESAVI/7812/04.10.07/2015). A total of 123 adult male Wistar rats weighing 210-350 g (RRID: RGD_5508396, Harlan/Envigo, Horst, The Netherlands) were used in the experiments. The animals were group housed under standard laboratory conditions in 12 h light/dark cycle with free access to food and water. The well-being of the animals was observed on a regular basis.

### Intrastriatal administration of viral vectors and 6-OHDA

All stereotaxic surgeries were performed under isoflurane anesthesia (4% induction and 2.5% maintenance) and carprofen (5 mg/kg, s.c.) was used as post-operative analgesic as previously described (26). For the viral vector injections, animals were randomly allocated to treatment groups. 4.5 μl of scAAV1-pre-α-pro-GDNF, scAAV1-pre-β-pro-GDNF or scAAV1-eGFP was equally distributed to three sites in the right striatum. AAV injections were carried out as previously described (27). The injection coordinates according to bregma were (1) A/P +1.6 L/M −2.8 D/V −6.0 from skull, (2) A/P 0.0 L/M −4.1 D/V −5.5 from skull, and 3) A/P-1.2 L/M −4.5 D/V −5.5 from skull (28). Injections were done in a 10° angle at a rate of 0.5 μl/min. The microinjection needle was kept in place for additional 5 min to avoid backflow of the solution (26). In the neuroprotection experiment 3 × 2 μg of 6-OHDA (Sigma Aldrich, St. Louis, MO) was injected to the same sites as the viral vectors 3 weeks later (**Figure 4A**).

### Tissue levels of GDNF

To assess the tissue levels of GDNF, 15 animals received 3 μl of scAAV1-pre-α-pro-GDNF (*n* = 5), scAAV1-pre-β-pro-GDNF (*n* = 5) or scAAV1-eGFP (*n* = 5) distributed evenly to the three striatal injection sites as described above. Three weeks later, animals were deeply anesthetized with pentobarbital (90 mg/kg, i.p., MebunatVet, Orion Pharma, Espoo, Finland) and decapitated. Brains were snap frozen in cold isopentane and stored at −70°C. The striatal samples were collected from the frozen brain and mechanically homogenized in lysis buffer (137 mM NaCl, 2.7 mM KCl, 8.1 mM Na_2_HPO_4_, 1% Igepal, 10% glycerol, 1:25 Complete Mini EDTA-free (Roche, Basel, Switzerland) and centrifuged at 5,000 × g for 5 min at +4°C. The GDNF levels were determined from the supernatants by commercial enzyme-linked immunosorbent assay (ELISA) kit according to the manufacturer's recommendations (Promega, Madison, WI).

### Behavioral assays

#### Cylinder test

**Figure 2:**
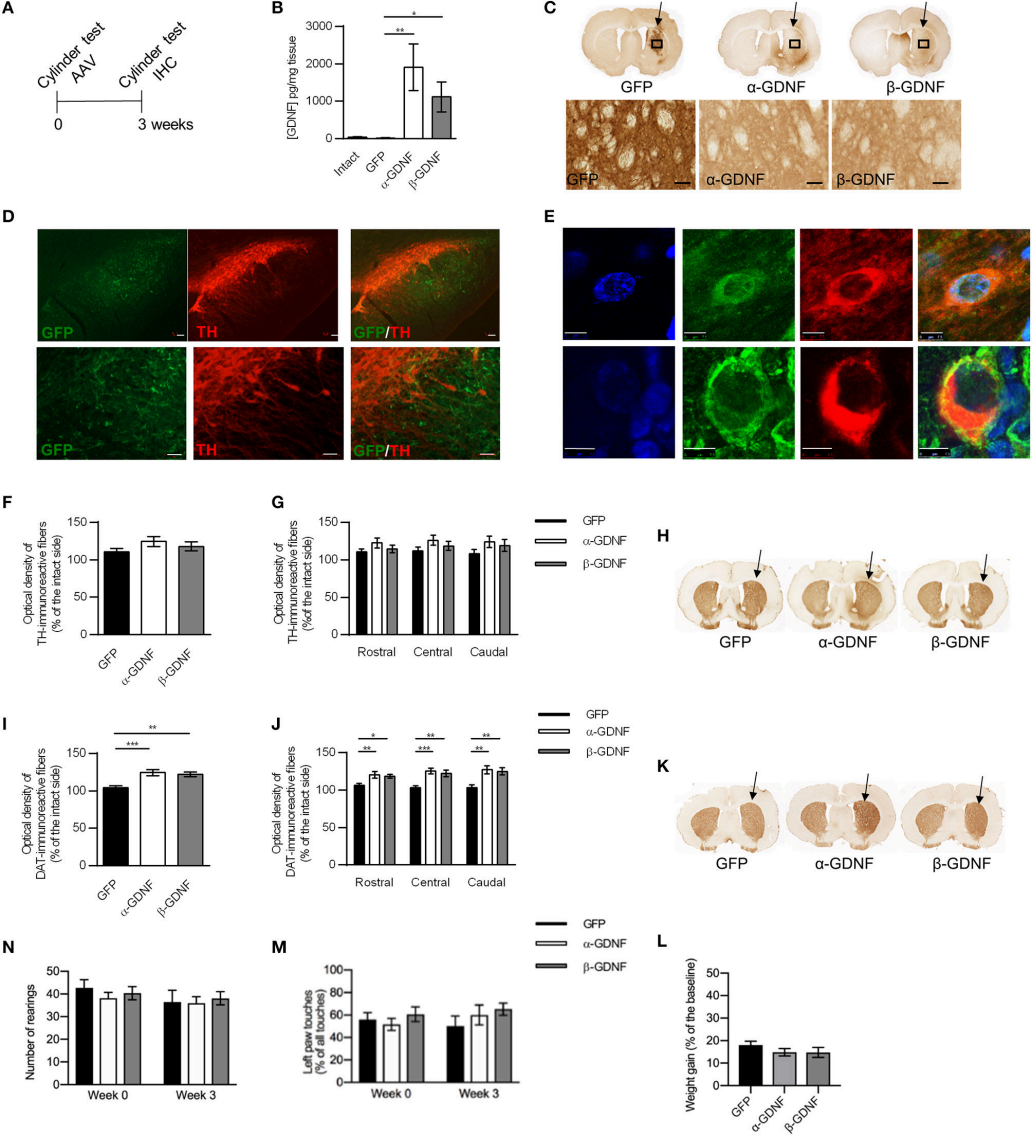
Effects of GDNF isoform overexpression on dopaminergic markers in non-lesioned striatum 3 weeks after AAV-injection. **(A)** Experimental design. **(B)** Overexpression levels of GDNF isoforms were confirmed with ELISA [Kruskal-Wallis test H(3) = 15.457, *p* = 0.001, followed by Bonferroni corrected Mann-Whitney U *post-hoc* test, ^**^p < 0.01, ^*^p < 0.05, *n* = 5 in each group]. **(C)** Representative images of GFP- and GDNF-stained striatal sections. Arrows point to the injected side. 40x magnification of the area is designated by the black box and scale bar is 50 μm. **(D)** GFP signal was observed in SN reticulata, but not in TH-immunoreactive cells in SNpc. Upper panels show 5x magnification with scale bar 100 μm, lower panels show 20x magnification with scale bar 50 μm. **(E)** Both GDNF isoforms co-localized with scgII-immunoreactive structures. Blue = dapi, green = scgII, red = GDNF (upper row alpha, lower row beta), scale bar 7.5 μm. **(F)** Optical density of striatal TH-immunoreactive fibers was similar in all treatment groups (GFP 111 ± 4%, α-GDNF 124 ± 7%, and β-GDNF 118 ± 6% of the intact side, *n* = 8–10 in each group) **(G)** Density of TH-immunoreactive fibers was at similar level in all treatment groups throughout the whole striatum (*n* = 8–10). **(H)** Representative images of TH-stained striatal sections. Arrows point to the injected side. **(I)** Overexpression of both GDNF isoforms increased the optical density of striatal DAT-immunoreactive fibers one-way ANOVA *F*_(2, 24)_ = 11.336, *p* < 0.001, Fisher's LSD *post-hoc* test α-GDNF vs. GFP *p* < 0.001 and β-GDNF vs. GFP *p* = 0.002, ^***^p < 0.001, ^**^p < 0.01, *n* = 8–10]. **(J)** The effects of GDNF isoforms were consistent throughout whole striatum [one-way ANOVA rostral *F*_(2, 24)_ = 5.315, *p* = 0.012, Fisher's LSD *post-hoc* analysis α-GDNF vs. GFP *p* = 0.005 and β-GDNF vs. GFP *p* = 0.026; central *F*_(2, 24)_ = 11.339, *p* < 0.0001, Fisher's LSD *post-hoc* analysis α-GDNF vs. GFP *p* < 0.0001 and β-GDNF vs. GFP *p* = 0.002; caudal: *F*_(2, 24)_ = 7.674, *p* = 0.003 Fisher's LSD *post-hoc* analysis α-GDNF vs. GFP *p* = 0.001 and β-GDNF vs. GFP *p* = 0.006, ^***^p < 0.001, ^**^p < 0.01, ^*^p < 0.05 *n* = 8–10]. **(K)** Representative images of DAT-stained striatal sections. Arrows point to the injected side. **(L,M)** Short-term overexpression of GDNF isoforms in non-lesioned striatum did not induce behavioral changes in the cylinder test, as measured by **(N)** vertical activity (baseline GFP 43 ± 4, α-GDNF 38 ± 3, and β-GDNF 40 ± 3 rearings, 3 weeks after scAAV GFP 37 ± 5, α-GDNF 36 ± 3, and β-GDNF 38±3 rearings) or **(M)** contralateral paw touches, (*n* = 8–10 in each group). **(L)** All animals gained weight in similar manner during the 3 weeks of the experiment (*n* = 15 in each group). Data is expressed as mean ± SEM.

**Figure 3:**
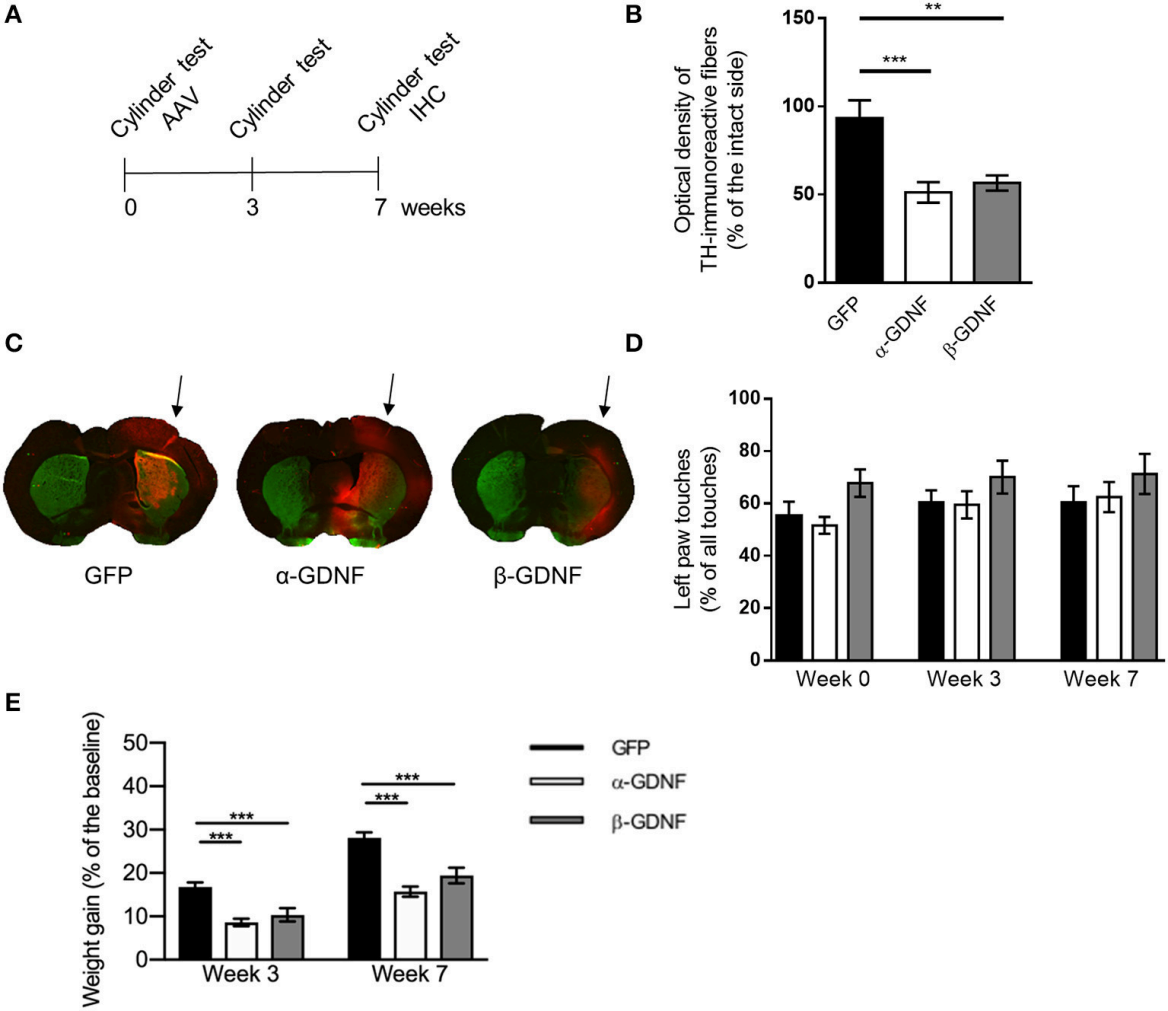
Effects of GDNF isoform overexpression on dopaminergic markers in non-lesioned striatum 7 weeks after AAV-injection. **(A)** Experimental design. **(B)** Optical density of striatal TH-immunoreactive fibers was significantly lower in both isoform treated groups compared to GFP [one-way ANOVA, *F*_(2, 28)_ = 10.56, *p* = 0.0004, followed by Bonferroni *post-hoc* test, ^***^p < 0.001, ^**^p < 0.01 *n* = 10–11]. **(C)** Representative images of TH- (green), GFP-, (red) and GDNF- (red) stained striatal sections from infrared analysis, arrows point to the injected side. **(D)** No significant changes in contralateral (left) paw touches were observed 3 or 7 weeks after injection of GDNF isoforms [one-way ANOVA *F*_(2, 28)_ = 0.7678, *p* = 0.4736]. Data is expressed as mean ± SEM. **(E)** Animals treated with either α- or β-GDNF isoform gained weight significantly less compared to GFP-treated animals, both 3 and 7 weeks after AAV-injections [two-way ANOVA treatment effect *F*_(2, 58)_ = 33.044, *p* < 0.0001; time effect *F*_(1, 58)_ = 5 2,966, *p* < 0.0001; treatment × time interaction *F*_(2, 58)_ = 0.358, *p* = 0.701]. 3 week time point 1-way ANOVA *F*_(2, 29)_ = 13.040, *p* < 0.0001, Fisher's LSD *post-hoc* test GFP vs. α-GDNF *p* < 0.001, GFP vs. β-GDNF vs. *p* = 0.001, and α-GDNF vs. β-GDNF *p* = 0.294. Seven week time point one-way ANOVA *F*_(2, 29)_ = 18.689, *p* < 0.0001, Fisher's LSD *post-hoc* test GFP vs. α-GDNF *p* < 0.0001, GFP vs. β-GDNF vs. *p* = 0.0001, and α-GDNF vs. β-GDNF *p* = 0.073, ^***^p < 0.001, *n* = 10 in each group.

**Figure 4:**
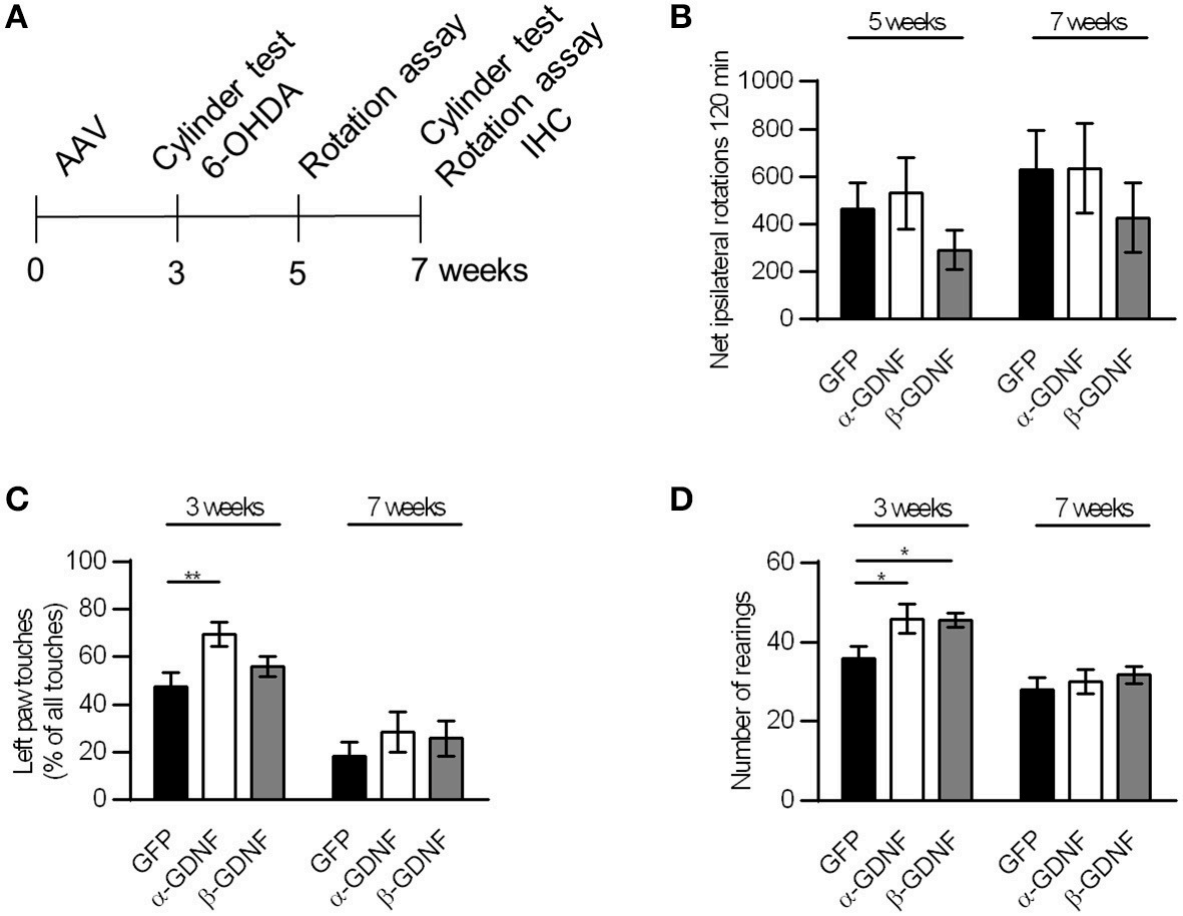
Neither GDNF isoform displayed neuroprotective effects in the rotational assay or the drug-free cylinder test. **(A)** Experimental design. **(B)** The rotational behavior was at similar level in all treatment groups (*n* = 15–16). **(C)** α-GDNF increased the use of contralateral paw in the pre-lesion cylinder test on week three [one-way ANOVA *F*_(2, 43)_ = 4.492, *p* = 0.017, followed by Fisher's LSD *post-hoc* test ^**^p < 0.01, *n* = 15–16], but the effect was abolished after 6-OHDA administration. **(D)** Both GDNF isoforms increased the exploratory activity of the animals before the lesion in the cylinder test [one-way ANOVA *F*_(2, 43)_ = 3.871, *p* = 0.028, followed by Fisher's LSD *post-hoc* test ^*^p < 0.05, *n* = 15–16 in each group]. Data is expressed as mean ± SEM.

Motor asymmetry was assessed with the cylinder test before viral vector administration and 3 and 7 weeks after the administration (Figures 2A, 3A). In the neuroprotection experiment the cylinder test was conducted 3 and 7 weeks after virus injection (before and 4 weeks after 6-OHDA injection) (Figure 4A). Freely moving rats were monitored for 5 min in a plexiglass cylinder (diameter 20 cm) under red light, and the contacts between forepaws and the cylinder wall were counted by a blinded observer. Placement of the whole palm on the cylinder wall to support the body while exploring was considered as a touch.

#### Rotation assay

In the neuroprotection experiment, the motor asymmetry was also measured with the d-amphetamine-induced rotation assay. The rotation assay was performed as previously described (26). In brief, the rotational behavior was monitored for 120 min after administration of d-amphetamine sulfate (2.5 mg/kg, s.c., Sigma Aldrich, St. Louis, MO) in automated rotation bowls (Med Associates, Inc., Fairfax, VT). Full 360° ipsilateral turns were given positive value.

### Tissue processing and immunohistochemistry

Three or seven weeks (neuroprotection experiment) after the virus injection, animals were anesthetized with pentobarbital (90 mg/kg, i.p., MebunatVet, Orion Pharma, Espoo, Finland) and transcardially perfused with phosphate buffered saline (PBS) and 4% paraformaldehyde (PFA) solution. Brains were removed and post-fixed overnight in 4% PFA at +4°C and transferred to sucrose series of 20 and 30% sucrose.

The brains were cut in a freezing microtome in 40 μm thick sections in series of six. Free-floating sections were stained as previously described (26). In brief, the sections were washed and treated with 0.3% hydrogen peroxide solution. For DAT staining, the sections were incubated in 10 mM citrate buffer, pH 6.0, at 80°C for 30 min. After incubation in the blocking solution (4% bovine serum albumin and 0.1% Triton X-100 in PBS) the sections were incubated with the primary antibody overnight at +4°C. Primary antibodies and the dilutions used in the studies are designated in Table 1. Next, the sections were incubated with biotinylated secondary antibodies (anti-rat, anti-mouse, or anti-rabbit, Vector Laboratories, Burlingame, CA) and the staining was reinforced with avidin-biotin-complex (Vector Laboratories, Burlingame, CA) and visualized with 3′, 3′diaminobenzidine. The stained sections were scanned with automated microscope slide scanner (Pannoramic 250 Flash II, 3D Histech, Budapest, Hungary).

**Table 1:** Antibodies and their dilutions used in immunohistochemistry.

**Antigen**	**Host**	**Producer**	**Cat#**	**RRID**	**Dilution**
Dopamine transporter (DAT)	Rat	Millipore	MAB369	AB_2190413	1:2,000
Glial cell line-derived neurotrophic factor (GDNF)	Goat	R & D systems	AF-212-NA	AB_2111398	1:3,000^a^ 1:1,000^b^
Green fluorescent protein (GFP)	Rabbit	Life technologies	A11122	N/A	1:2,000
Tyrosine hydroxylase (TH)	Mouse	Millipore	MAB318	AB_2201528	1:2,000
Secretogranin II	Mouse	Abcam	ab20246	AB_445463	1:500

a *In expression pattern studies*.

b *Confocal microscopy and infrared analysis*.

To detect scAAV1 transduction pattern in the SN, immunofluorescence staining was carried out for the sections. The sections were incubated in the blocking solution (4% bovine serum albumin and 0.1% Triton X-100 in PBS), followed by incubation with primary antibody (anti-TH, Table 1) overnight at +4°C. After washing, the sections were incubated with Alexa 568-conjugated goat-anti-mouse secondary antibody (1:300, ThermoFisher Scientific, Waltham, MA) and mounted on microscope slides. GFP signal was visible without immunofluorescence staining.

For the confocal microscopy, the striatal sections were incubated with blocking solution for 1 h followed by 1 h incubation with the first primary antibody (ScgII, Table 1) at RT. After this, the second primary antibody (anti-GDNF) was added and the sections were incubated at +4°C overnight. The following day, sections were incubated with Alexa 488-conjugated donkey-anti-mouse secondary antibody (1:500, ThermoFisher Scientific, Waltham, MA) antibody for 15 min and then for 1 h after the addition of Alexa 568-conjugated donkey-anti-goat secondary antibody (1:500, ThermoFisher Scientific, Waltham, MA) at RT. Sections were mounted in PBS, allowed to dry overnight, washed in ddH_2_O, allowed to dry o/n and subsequently coverslipped using Vectashield HardSet Antifade Mounting Medium with DAPI (H-1500; Vector Labs, Burlingame, CA).

For infrared analysis, the sections were incubated with blocking solution for 1 h followed by 1 h incubation with the primary antibody for anti-TH at RT. After this, the second primary antibody (anti-GFP or anti-GDNF, Table 1) was added and the sections were incubated at +4°C overnight. Next day, sections were incubated in IRDye® 800CW secondary antibody for 15 min and then for 1 h after the addition of the other secondary antibody, anti-Goat or anti-Rabbit IRDye® 680RD (All secondary antibodies 1:2,000, LI-COR Biosciences, Lincoln, NE) at RT. Before mounting, the sections were rinsed with ddH_2_O for 5 min at RT.

### Confocal microscopy

Slides were imaged using a Leica TCS SP5 confocal microscope (CLSM; Leica Microsystems, Buffalo Grove, IL) through a 63 × oil-immersion objective. The brightness/contrast of the image taken with Laser-405 (DAPI) was adjusted by ImageJ for optimal visual display.

### Estimation of optical density of TH- and DAT-immunoreactive fibers in the striatum

The density of TH- and DAT-immunoreactivity was measured from six adjacent sections with ImagePro software (Media Cybernetics, Inc., Rockville, MD) by a blinded observer. Corpus callosum was used as a background to correct the values. The data are presented as a percentage of the intact side.

In the infrared assay, the sections were scanned with Odyssey Infrared Imaging System (LI-COR Biosciences, Lincoln, NE) with 42-micron resolution. The TH optical densities from the injected and non-injected (intact) side of four striatal sections per animal were measured using the Odyssey Infrared Imaging System software. Background optical density was measured from the cortex or corpus callosum depending on the integrity of the section. The density of TH-immunoreactive fibers was assessed by subtracting the background intensity values and normalizing the injected side to the optical density of the intact side. The data are presented as a percentage of the intact side.

### Estimation of number of TH-immunoreactive cells in the SNpc

The number of TH-immunoreactive cells in the SNpc was determined with Matlab (RRID: SCR_001622, MathWorks, Kista, Sweden) as previously described (26) by a blinded observer. Images taken with whole slide scanner (Pannoramic 250 Flash II, 3D Histech, Budapest, Hungary, with 20x objective) from six adjacent nigral sections were analyzed. The data are presented as a percentage of the intact side.

### Statistics

Results are given as mean ± SEM. Statistical analysis was performed with SPSS (RRID: SCR_002865, IBM, Armonk, NY) or Prism version 6.01 (GraphPad Software, Inc., La Jolla, San Diego, CA). Differences between treatment groups were assessed with one-way analysis of variance (ANOVA) or two-way ANOVA and if significant, followed by Fisher's Least Significant Difference (LSD) or Bonferroni *post-hoc* analysis (7 week overexpression experiment). In cases of non-homogenous variances (ELISA assay), Kruskal-Wallis analysis of variance followed by Bonferroni corrected Mann-Whitney U *post-hoc* was conducted. A difference was considered to be significant at *p* ≤ 0.05.

## Results

### Overexpression of GDNF in the non-lesioned striatum

A scAAV-vector encoding pre-α-pro-GDNF (α-GDNF), pre-β-pro-GDNF (β-GDNF), or green fluorescent protein (GFP, as a control) was injected into three sites in the non-lesioned striatum. The level of GDNF overexpression was determined with ELISA 3 weeks after the gene delivery. The infusion of scAAVs produced a marked overexpression of GDNF in the striatum (Figure 2B). The level of GDNF in the intact (contralateral) side was 40 ± 8 pg/mg tissue, in scAAV-GFP-treated side 15 ± 6 pg/mg tissue, in scAAV-α-GDNF-treated side 1,906 ± 629 pg/mg tissue, and in scAAV-β-GDNF-treated side 1,115 ± 402 pg/mg tissue (GFP vs. α-GDNF *p* = 0.005, GFP vs. β-GDNF *p* = 0.017, and α-GDNF vs. β-GDNF *p* = 0.465, Figure 2B).

Although the ELISA results showed robust GDNF overexpression in the striatum, immunohistochemistry was also applied to explore the protein distribution along the nigrostriatal tract. Since GFP is retained inside the cells, it had more restricted staining pattern in the striatum (Figure 2C). In contrast, GDNF is a secretory protein (1) and the staining pattern was widely spread, covering most of the striatum of the injected side. Minimal immunoreactivity was observed on the contralateral, non-injected side. To determine whether scAAV1 transduces post-synaptic striatal neurons, pre-synaptic dopamine neurons, or both we carried out immunofluorescence staining for TH and compared it to GFP. GFP expression in the SN reticulata was not in TH-immunoreactive fibers or TH-immunoreactive cells of the SNpc (Figure 2D). This staining pattern suggests that striatal delivery of scAAV1 does not transduce nigrostriatal dopaminergic neurons, but nigral gene expression is due to transduction of striatal medium spiny projection neurons. *In vitro* β-GDNF has been shown to co-localize mostly with the scgII signal in the cells, unlike α-GDNF (17). In contrast, we found that overexpression with AAVs under the CMV promoter *in vivo* both α-GDNF and β-GDNF were found to be co-localized with the scgII-signal (Figure 2E). Moreover, both isoforms were ubiquitously expressed in cell bodies, and no specific sub-localization patterns were observed.

### Overexpression of GDNF isoforms do not alter the density of TH-immunoreactive fibers but increases the density of DAT-immunoreactive fibers in the non-lesioned striatum 3 weeks after scAAV delivery

Since GDNF has been shown to regulate the markers for dopaminergic phenotype, we next studied the effects of α-GDNF or β-GDNF overexpression on the dopaminergic markers TH and dopamine transporter (DAT) in non-lesioned striatum 3 weeks after the scAAV administration. GDNF overexpression did not alter the striatal TH optical density. Thus, the optical densities of striatal TH-immunoreactive fibers were similar in all treatment groups (Figures 2F,H). We divided sections into three categories: rostral, central, and caudal, each containing two adjacent sections, to analyze the TH optical density along the rostrocaudal axis in the striatum. The density of TH-immunoreactive fibers was at the same level in all three striatal areas for all treatment groups (Figure 2G). In contrast, the density of DAT-immunoreactive fibers was increased in α- and in β-GDNF-treated animals compared to GFP-treated animals (α-GDNF vs. GFP p < 0.001 and β-GDNF vs. GFP *p* = 0.002, Figures 2I,K). Furthermore, the effect of GDNF isoforms on DAT-immunoreactive fiber density was increased in all sections along the rostro-caudal axis in the striatum [two-way ANOVA treatment effect *F*_(2, 72)_ = 23.285, *p* < 0.0001; site effect *F*_(2, 72)_ = 0.490, *p* = 0.615; treatment × site effect *F*_(4, 72)_ = 0.588, *p* = 0.672, Figure 2J).

Overexpression of GDNF isoforms did not change the behavior of the animals in the cylinder test. The vertical activity of the animals remained on the same level 3 weeks after scAAV-administration compared to baseline measured before the viral vector delivery (Figure 2L). The use of the contralateral paw was at similar level in all treatment groups both before viral vectors were administered and 3 weeks later (Figure 2M). Furthermore, animals gained weight comparably by the 3 week time point (Figure 2N).

### Overexpression of GDNF isoforms decreases the density of TH-immunoreactive fibers in the non-lesioned striatum 7 weeks after viral vector delivery

The effect of GDNF isoform overexpression on striatal TH-immunoreactivity was assessed also 7 weeks after viral vector delivery (Figures 3A–C). At this time point, there was a significant decrease in the density of TH-immunoreactive fibers in the non-lesioned striata of both α-GDNF and β-GDNF-treated animals (GFP vs. α-GDNF *p* = 0.0006 and GFP vs. β-GDNF *p* = 0.0026). There was no statistically significant difference between the GDNF isoform groups.

Unlike in the shorter (3 week) overexpression study, no differences in the use of contralateral paw was observed 7 weeks after injections in the cylinder test (Figure 3D). The use of the contralateral paw was at a similar level in all treatment groups, before viral vectors were administered, 3 weeks as well as 7 weeks after AAV injections.

Interestingly, non-lesioned GDNF-treated animals gained less weight than non-lesioned GFP-treated animals (Figure 3E). Three weeks after viral vector administration GFP-treated animals had gained weight 17 ± 1%, α-GDNF 9 ± 1% and β-GDNF 10 ± 1% compared to their initial weight (GFP vs. α-GDNF *p* < 0.001, GFP vs. β-GDNF vs. *p* = 0.001). Four weeks later, 7 weeks after the viral vector delivery, GFP-treated animals had gained weight 28 ± 1%, α-GDNF 16 ± 1%, and β-GDNF 19 ± 2% of their initial weight (GFP vs. α-GDNF *p* < 0.0001, GFP vs. β-GDNF vs. *p* = 0.0001, and α-GDNF vs. β-GDNF *p* = 0.073, Figure 3E).

### GDNF splice isoforms protect TH-immunoreactive cells in SNpc with no behavioral correlates

The neuroprotective effects of GDNF splice isoforms were tested in the 6-OHDA partial lesion model. scAAV encoding either α-GDNF, β-GDNF, or GFP was administered into three sites in the striatum, and 3 weeks later 6 μg of 6-OHDA was evenly distributed (3 × 2 μg) to the same sites as the viral vector. The effects were evaluated with the d-amphetamine-induced rotation assay 5 and 7 weeks after scAAV-injection (2 and 4 weeks after lesioning, respectively), as well as with the drug-free cylinder test 3 and 7 weeks after scAAV-injection (before and 4 weeks after lesioning, respectively, Figure 4A). Amphetamine-induced rotational behavior was at similar level in all treatment groups on week five and on week seven (Figure 4B). Two-way ANOVA did not show significant effects in rotational behavior [treatment effect *F*_(2, 92)_ = 1.333, *p* = 0.269; time effect *F*_(1, 92)_ = 1.270, *p* = 0.263; treatment × time *F*_(2, 92)_ = 0.020, *p* = 0.980].

In the pre-lesion cylinder test on week 3, α-GDNF-treated animals showed increased use of contralateral (left) paw (GFP vs α-GDNF *p* = 0.005, Figure 4C). 6-OHDA injection reduced the use of the contralateral paw in all groups to the same level [two-way ANOVA treatment effect *F*_(2, 86)_ = 3.215, *p* = 0.045; 6-OHDA effect *F*_(1, 86)_ = 41.803, *p* < 0.0001; treatment × 6-OHDA interaction *F*_(2, 86)_ = 0.545, *p* = 0.582). Even though only α-GDNF showed an effect in the spontaneous use of paws, both isoforms increased the exploratory activity of the animals on week three, seen as an increase in the amount of rearings (GFP vs α-GDNF *p* = 0.019 and GFP vs. β-GDNF *p* = 0.024, Figure 4D). Four weeks after 6-OHDA administration the exploratory activity was reduced to the same level in all treatment groups [two-way ANOVA treatment effect *F*_(2, 86)_ = 3.406, *p* = 0.038; 6-OHDA effect *F*_(1, 86)_ = 29.071, *p* < 0.0001; treatment × 6-OHDA interaction *F*_(2, 86)_ = 1.130, *p* = 0.328].

**Figure 5:**
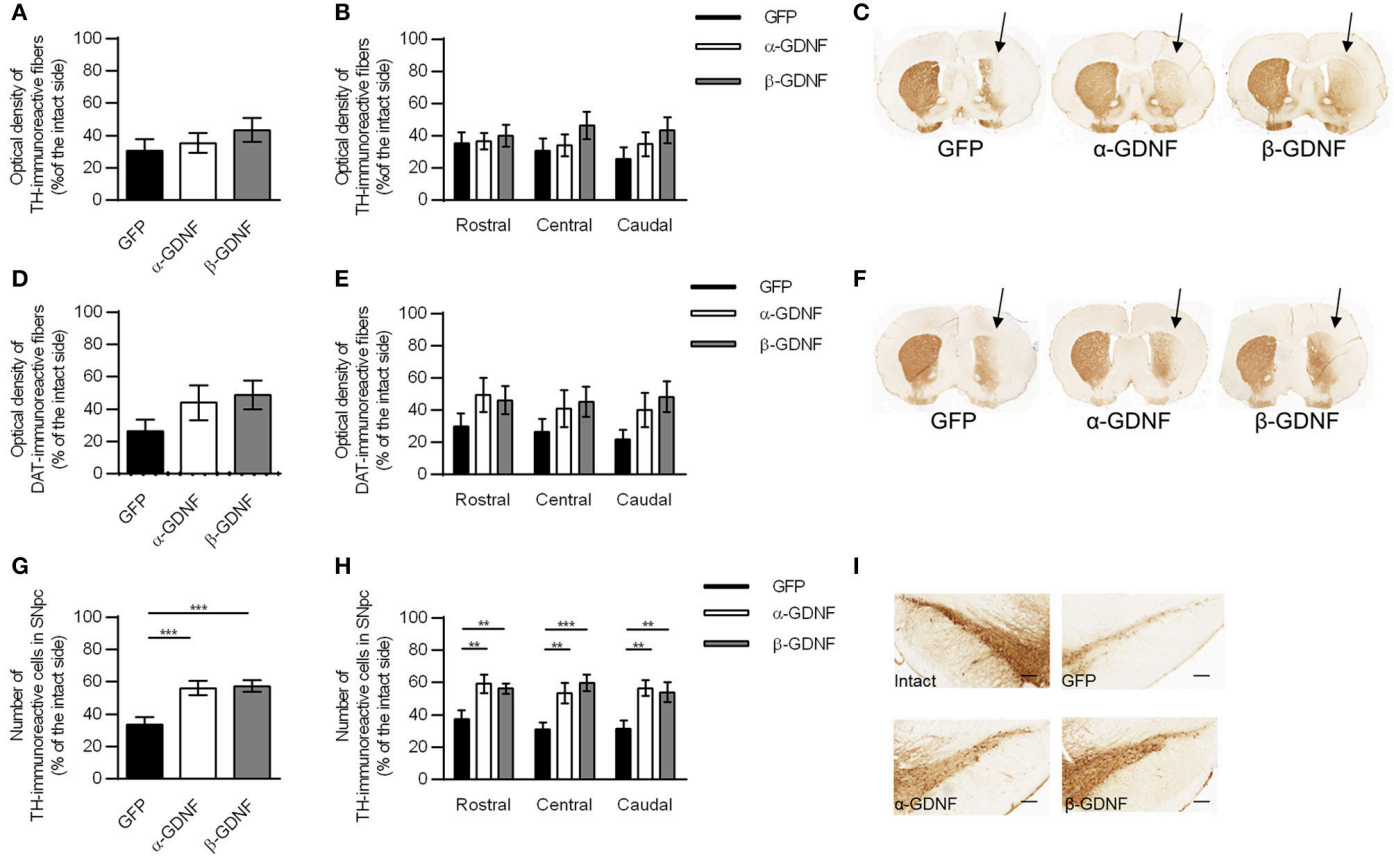
Immunohistochemistry revealed both isoforms to protect TH-immunoreactive cells in the SNpc. **(A)** Optical density of striatal TH-immunoreactive fibers was decreased in all treatment groups 4 weeks after 6-OHDA administration (*n* = 14–15). **(B)** The density of TH-immunoreactive fibers was at the same level through the whole rostro-caudal axis of striatum (*n* = 14–15). **(C)** Representative images of striatal TH-immunoreactivity. **(D)** Optical density of striatal DAT-immunoreactive fibers [one-way ANOVA *F*_(2, 29)_ = 1.815, *p* = 0.181, *n* = 10–11]. **(E)** Optical density of DAT-immunoreactive fibers was increased in GDNF-treated animals throughout whole striatum [two-way ANOVA treatment effect *p* = 0.015, One-way ANOVA rostral *F*_(2, 28)_ = 1.358, *p* = 0.274; central: *F*_(2, 28)_ = 1.045, *p* = 0.375; caudal: *F*_(2, 29)_ = 2.343, *p* = 0.114, *n* = 10–11]. **(F)** Representative images of striatal DAT-immunoreactivity. **(G)** Both GDNF isoforms increased the number of TH-immunoreactive cells in the SNpc [one-way ANOVA *F*_(2, 42)_ = 8.828, *p* < 0.001, followed by Fisher's LSD analysis, ^***^p < 0.001, *n* = 14–16]. **(H)** The effect was consistent throughout the whole SNpc [One-way ANOVA rostral *F*_(2, 42)_ = 6.004, *p* = 0.005, Fisher's LSD *post-hoc* analysis α-GDNF vs. GFP *p* = 0.003 and β-GDNF vs. GFP *p* = 0.008; central *F*_(2, 41)_ = 8.784, *p* = 0.001, Fisher's LSD *post-hoc* analysis α-GDNF vs. GFP *p* = 0.004 and β-GDNF vs. GFP *p* < 0.0001; caudal *F*_(2, 41)_ = 7.214, *p* = 0.002 Fisher's LSD *post-hoc* analysis α-GDNF vs. GFP *p* = 0.001 and β-GDNF vs. GFP *p* = 0.004, ^**^p < 0.01, ^***^p < 0.001, *n* = 14–16]. **(I)** Representative images of TH-immunoreactivity in the SN. Scale bar 200 μm. Data expressed as mean ± SEM.

The density of TH-immunoreactive fibers in the striatum was at the same level in all treatment groups (Figures 5A,C) 4 weeks after 6-OHDA administration. The density of TH-immunoreactive fibers was similar over the whole striatum in all groups (Figure 5B). The density of DAT-immunoreactive fibers was increased in GDNF-treated groups, but the difference did not reach statistical significance (Figures 5D–F). When the striatal DAT-immunoreactivity was analyzed in more detail throughout the striatum, two-way ANOVA revealed a significant treatment effect [treatment effect *F*_(2, 85)_ = 4.388, *p* = 0.015; site effect *F*_(2, 85)_ = 0.272, *p* = 0.762; treatment × site interaction *F*_(4, 85)_ = 0.130, *p* = 0.971; Figure 5E].

In the SNpc both GDNF isoforms protected and rescued TH-immunoreactive cells (GFP vs. α-GDNF *p* = 0.001 and GFP vs. β-GDNF *p* < 0.001, Figures 5G,I). The difference between GDNF-treated animals and GFP-treated animals was consistent in all three analyzed areas [two-way ANOVA treatment effect *F*_(2, 124)_ = 21.493, *p* < 0.001; site effect *F*_(2, 124)_ = 0.388, *p* = 0.679; treatment × site interaction *F*_(4, 124)_ = 0.352, *p* = 0.842, Figure 5H].

### Striatal overexpression of GDNF isoforms induces sprouting of TH- and DAT-immunoreactive fibers in globus pallidus

**Figure 6:**
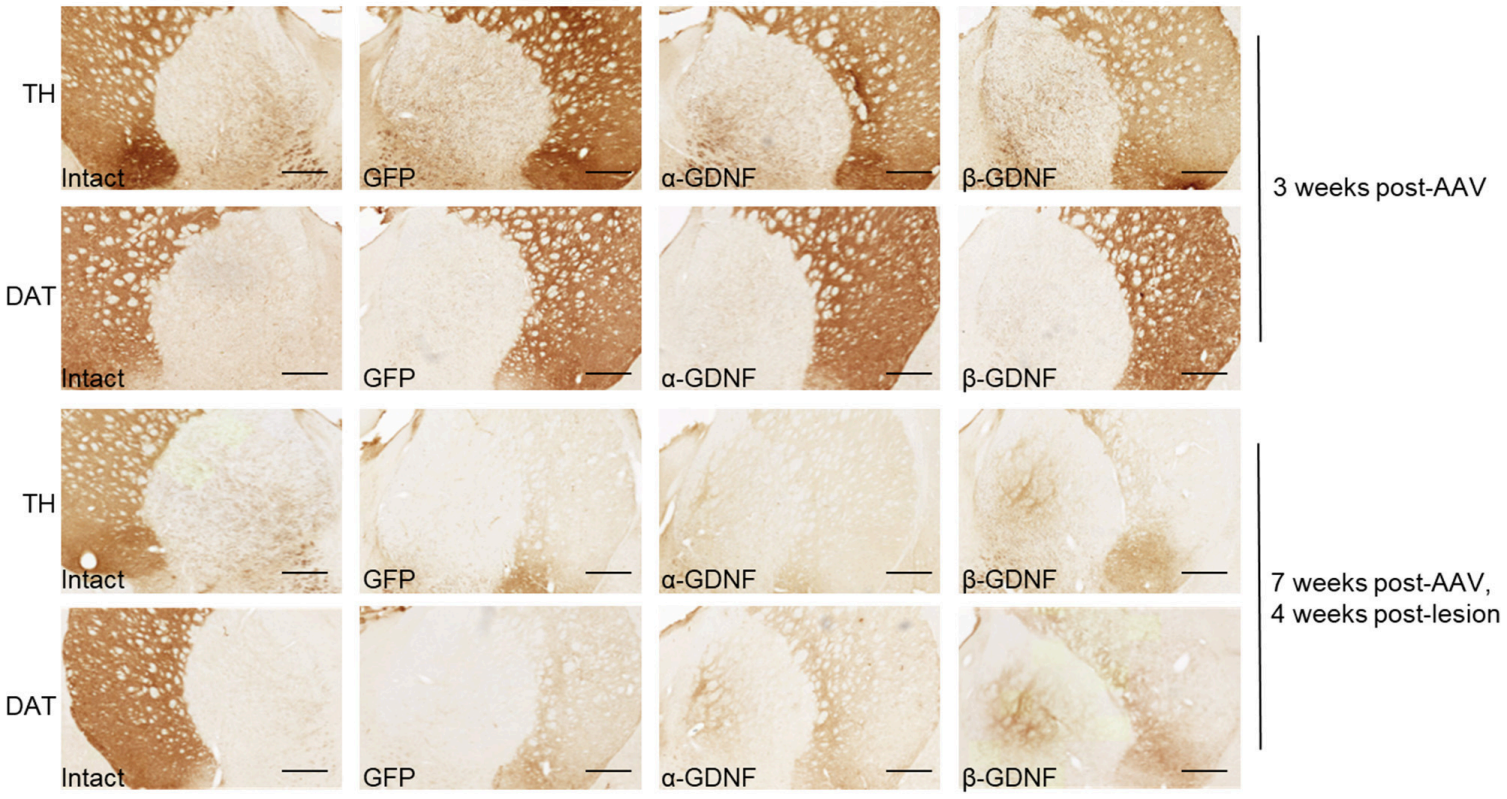
Both GDNF isoforms induced sprouting of TH- and DAT-immunoreactive fibers in globus pallidus. Representative images from intact side, GFP, α-GDNF, and β-GDNF treated animals. Scale bar is 500 μm.

Administration of exogenous GDNF has been shown to induce sprouting around the nigrostriatal pathway (29–32). In non-lesioned animals, sprouting was not observed 3 weeks after viral vector injection (Figure 6). Instead, the sprouting of TH- and DAT-immunoreactive fibers in the globus pallidus (GP) was detected 7 weeks after virus injection, 4 weeks after 6-OHDA injection, in both α- and β-GDNF-treated groups (Figure 6). In contrast, 6-OHDA injection cleared the TH- and DAT-immunoreactivity completely from the GP of GFP-treated animals.

## Discussion

Until now, very little has been known about the biology of the shorter β-GDNF isoform and its functions in the adult mammalian brain. We compared the effects of full-length α-GDNF and the shorter β-GDNF splice isoforms in non-lesioned animals and in the partial 6-OHDA rat model of PD. Both GDNF splice isoforms were overexpressed with their native pre-pro-sequences (pre-α-pro-GDNF and pre-β-pro-GDNF) in striatum using scAAV1 vectors. We found that in the non-lesioned striatum, both isoforms increased the density of DAT-immunoreactive fibers and decreased the density of TH-immunoreactive fibers. In the neuroprotection assay, both α-GDNF and β-GDNF overexpression increased the number of TH-immunoreactive cells after 6-OHDA-induced degeneration.

GDNF is produced as a precursor protein, pre-pro-GDNF, and proteolytically cleaved to mature GDNF in endoplasmic reticulum and secretory vesicles (1, 17). Although the pro-region is not necessarily needed for secretion of GDNF, it has been suggested to have a role in the protein folding and secretion (18, 33). In addition, the full-length pro-region of α-GDNF contains an 11 amino acids long peptide, dopamine neuron stimulating peptide-11 or brain excitatory peptide (14, 34, 35), which has both neurotrophic and neuroprotective properties *in vitro* and *in vivo* (35). GDNF produced in mammalian cells has been shown to be more stable than GDNF produced in *E. coli* (18), possibly due to posttranslational modifications. These findings support the consideration of pro-GDNF for future gene and protein-based therapies using GDNF.

Administration of AAVs encoding the GDNF isoforms to the striatum is in accordance with the target derived hypothesis of neurotrophic factors. This paradigm is also warranted by reports that the receptors for GDNF signaling, GFRα1 and Ret are expressed in the midbrain dopamine neurons (36, 37). The exact mechanism of GDNF's neuroprotective effects remains unknown, but the striatal delivery of GDNF might affect the neuronal targets of the nigrostriatal pathway, inducing axonal sprouting and re-innervation (7, 30). This results in functional recovery, despite only partially protecting nigral TH-immunoreactive cell bodies. However, protection of nigral TH-immunoreactive cells without beneficial effect on behavior has been reported (38). On the other hand, nigral administration of GDNF prior to 6-OHDA provides almost complete protection of TH-immunoreactive cell bodies without functional recovery (7, 30). This lack of functional recovery might be due to the lack of sufficient axonal growth response and re-innervation of the lesioned striatum at the time of analysis (6, 7, 30, 31). Recent work demonstrates the importance of Ret in mediating neuroprotective and neurorestorative effects of GDNF (39). In addition, although endogenous GDNF is not required for survival midbrain dopamine neurons (40), increasing concentrations of endogenous GDNF at its native locus is neuroprotective (41).

In our experiments the striatal delivery of the GDNF gene before 6-OHDA administration neither isoform was able to attenuate the acute effects of striatal 6-OHDA but protected the nigral TH-immunoreactive cells partially from degeneration. The 6-OHDA lesion used in the experiment produced rather severe, 67% loss of TH-immunoreactive cells in the SNpc and 69% loss of TH-immunoreactive fibers in the striatum. The robust lesion might partly explain the lack of behavioral recovery, the level of GDNF overexpression wasn't sufficient to protect the nerve terminals from degeneration. In the rotation assay, β-GDNF treatment showed a tendency for initial protective effect 2 weeks after 6-OHDA lesion. Whether this mild, albeit not significant effect was due to 6-OHDA and/or amphetamine-induced secretion of β-GDNF, remains to be elucidated. However, the lack of functional effects might also be due to short follow-up period, 4 weeks after 6-OHDA injection, since Kirik and colleagues (7) have shown the behavioral effects to be detectable at earliest 7 weeks post-lesion in the cylinder and rotation assays.

Previous studies have shown that long-term overexpression of GDNF can cause changes in behavior and dopamine phenotype, and long-term high-expression of GDNF may not provide optimal neuroprotective effect (11, 42). In the pre-lesion cylinder test, α-GDNF-treated animals used their contralateral paw more compared to GFP- or β-GDNF-treated animals. Additionally, both GDNF splice isoforms increased the activity of the animals in the pre-lesion cylinder test. This is in line with earlier studies, where GDNF increased the locomotor activity of the animals (7, 43, 44). Exogenous GDNF has been shown to initially increase TH expression (43) and activity (7, 45), as well as the level of dopamine (43, 44) and dopamine turnover (7, 43). On the other hand, long-lasting overexpression of GDNF has been documented to downregulate TH expression in both lesioned and non-lesioned rat striatum (32, 46–49). Our observations are in line with these previously published studies, as downregulation of striatal TH was observed after 7 weeks of overexpression, but not in earlier, 3 week time point. Time-dependent downregulation and associated decrease in enzymatic activity can be due to feedback regulation after long-term dopamine neuron activity (49–51). Interestingly, overexpression of both α- and β-GDNF increased the density of striatal DAT-immunoreactive fibers in non-lesioned striatum after 3 weeks. While the long-term effects of GDNF on striatal DAT expression are still unclear, there seems to be dose-dependence, where lower doses of GDNF do not affect DAT expression, but higher doses downregulate DAT expression (52). Moreover, GDNF has been suggested to regulate DAT activity by increasing dimerization and protein-protein interactions (41, 51, 53). Downregulation of TH might be a species-specific phenomenon, as it has not been detected in non-human primates treated with viral vectors encoding GDNF (10, 54–60). Instead, TH-immunoreactivity is increased in the putamen of naïve non-human primates after GDNF-treatment (54, 55, 58). Also, these changes on dopamine phenotypic markers can be one explanation why we did not observe robust neuroprotective effects on striatal fibers.

As reported here and previously by others (29–32, 61) striatal administration induces loss of GP-passing fibers and striatal administration of GDNF induces sprouting of dopaminergic fibers in rostral GP and entopeduncular nucleus. In the rostral GP TH-immunoreactive fibers can be roughly divided to two different categories, thick and thin fibers. The thick fibers are more likely to represent the dopaminergic projections from SNpc to striatum passing through GP and the thinner TH-immunoreactive fibers direct dopamine afferent projections to the GP (61, 62). Besides sprouting of TH-immunoreactive fibers, we also observed sprouting of DAT-immunoreactive fibers in GP, suggesting axonal sprouting toward the striatum. Whereas this sprouting is considered to be a more beneficial phenomenon, nigral administration of GDNF induces sprouting around SN and along the nigrostriatal tract, which can be detrimental to the animals and even mask the beneficial effects of GDNF (7, 31).

In addition to affecting the behavior and dopaminergic phenotype, GDNF overexpression has been reported to induce weight loss in rats (45, 63). Long-term overexpression of GDNF isoforms in non-lesioned striatum slowed down the weight gain of animals. Though in the initial 3 week treatment we did not observe differences in the weight gain, subsequent 7 week treatment experiment showed a significant reduction in the weight gain for GDNF treated group both at 3 and 7 weeks post-treatment. One possible explanation for this is the difference in the initial weight of the animals. The long-term overexpression experiment was started with animals with average weight 321 g, whereas the short-term experiment was started with animals weighing 281 g on average. The conclusion from this experiment is that there is no difference between the isoforms on the weight gain.

The amounts of GDNF protein used in the clinical trials have been suggested to be excessive (18). In our study, both isoforms were overexpressed in a level comparable to previously published *in vivo* studies using viral vectors (7, 32, 47). However, the level of α-GDNF protein was higher than the level of β-GDNF. A similar phenomenon was reported when the GDNF splice variants were overexpressed in the brain using DNA nanoparticles (64). Moreover, in human brain the expression level of α-GDNF mRNA is higher compared to β-GDNF mRNA (14).

Selection of the vector construct does not only affect the expression level of the transgene, but also the localization of the transgene expression. In contrast to differences in the intracellular localization of the isoforms *in vitro* (17), *in vivo* both isoforms seemed to co-localize with scgII-positive secretory vesicles but were also present in the scgII-negative areas. This discrepancy might be due to the used cytomegalovirus promoter in the vector construct. A more specific promoter should be chosen to mimic the endogenous expression and localization patterns. Furthermore, the titer should be optimized to target scgII-positive vesicles specifically and to avoid over-saturation of the vesicles. In addition, to mimic the expression pattern of endogenous GDNF in striatum, the expression should be targeted to parvalbumin-positive interneurons (65).

To summarize, we compared the effects of the major GDNF splice isoforms, α-GDNF and β-GDNF, in non-lesioned striatum and in a partial 6-OHDA lesion model of PD. Studies with β-GDNF are of interest, since many of the GDNF's aforementioned effects are suggested to be dose-dependent. The differentially regulated secretion yet similar neuroprotective effects of β-GDNF compared to α-GDNF make β-GDNF an interesting candidate for PD therapy. Further studies are first needed to establish optimal gene delivery and therapeutic efficacy of pre-pro-β-GDNF.

## Author contributions

A-MP performed major components of the experiments, collected and analyzed the data and drafted the initial manuscript. IP conducted experiments, collected and analyzed data and edited the manuscript. AT, MK, MV, SB, and CR performed experiments and edited the manuscript. AD, BH, RT, LN, MS, and MA conceptualized the study and edited the manuscript. All authors have reviewed and approved the submitted version of the manuscript.

### Conflict of interest statement

The authors declare that the research was conducted in the absence of any commercial or financial relationships that could be construed as a potential conflict of interest.

## Abbreviations

6-OHDA: 6-hydroxydopamine
α-GDNF: pre-α-pro-GDNF
β-GDNF: pre-β-pro-GDNF
ANOVA: analysis of variance
DAT: dopamine transporter
GDNF: glial cell line-derived neurotrophic factor
GFP: green fluorescent protein
GP: globus pallidus
IHC: immunohistochemistry
PD: Parkinson's disease
scAAV: self-complementary adeno-associated virus
scgII: secretogranin II
SEM: standard error of mean
SNpc: substantia nigra pars compacta
TH: tyrosine hydroxylase.
